# Nitrogen-Use Efficiency, Nitrous Oxide Emissions, and Cereal Production in Brazil: Current Trends and Forecasts

**DOI:** 10.1371/journal.pone.0135234

**Published:** 2015-08-07

**Authors:** Marcel Viana Pires, Dênis Antônio da Cunha, Sabrina de Matos Carlos, Marcos Heil Costa

**Affiliations:** 1 Departamento de Economia Rural, Universidade Federal de Viçosa, Viçosa, Minas Gerais, Brazil; 2 Departamento de Engenharia Agrícola, Universidade Federal de Viçosa, Viçosa, Minas Gerais, Brazil; Agricultural Research Service, UNITED STATES

## Abstract

The agriculture sector has historically been a major source of greenhouse gas (GHG) emissions into the atmosphere. Although the use of synthetic fertilizers is one of the most common widespread agricultural practices, over-fertilization can lead to negative economic and environmental consequences, such as high production costs, depletion of energy resources, and increased GHG emissions. Here, we provide an analysis to understand the evolution of cereal production and consumption of nitrogen (N) fertilizers in Brazil and to correlate N use efficiency (NUE) with economic and environmental losses as N_2_O emissions. Our results show that the increased consumption of N fertilizers is associated with a large decrease in NUE in recent years. The CO_2_ eq. of N_2_O emissions originating from N fertilization for cereal production were approximately 12 times higher in 2011 than in 1970, indicating that the inefficient use of N fertilizers is directly related to environmental losses. The projected N fertilizer forecasts are 2.09 and 2.37 million ton for 2015 and 2023, respectively. An increase of 0.02% per year in the projected NUE was predicted for the same time period. However, decreases in the projected CO_2_ eq. emissions for future years were not predicted. In a hypothetical scenario, a 2.39% increase in cereal NUE would lead to $ 21 million savings in N fertilizer costs. Thus, increases in NUE rates would lead not only to agronomic and environmental benefits but also to economic improvement.

## Introduction

Agricultural practices have historically been a major source of greenhouse gas (GHG) emissions into the atmosphere [1]. The Agriculture, Forestry, and Other Land Use (AFOLU) sector is responsible for just under a quarter (~10–12 Gt CO_2_ eq. yr^-1^) of all anthropogenic GHG emissions, mainly from deforestation and agricultural emissions from livestock, soil and nutrient management [2]. According to Rosenzweig and Tubiello [3], out of all GHGs released annually into the atmosphere by human activities, the AFOLU sector is responsible for approximately a quarter of carbon dioxide (CO_2_) emissions through deforestation, depletion of soil organic C, use of machinery and manufactured fertilizers; half of methane (CH_4_) emissions via livestock and rice cultivation; and three quarters of nitrous oxide (N_2_O) emissions through fertilizer application and animal waste management. In Brazil, agriculture and livestock accounted for one-third of the gross national emissions in 2010 [4].

Agricultural systems around the world have been increasingly intensified, with the objective of meeting the growing demand for food, feed, fuel and fiber [5]. According to the Food and Agriculture Organization of the United Nation’s projections [6], cereal production will increase by 60% from 2000 to 2050. Thus, the use of manufactured fertilizers will increase over the coming years to cope with these changes [7]. Recent projections indicate an increase in global fertilizer consumption of approximately 69 million ton in 2030, and the increased use of nitrogen (N) fertilizers is responsible for 67% of such amount [8]. However, the excessive and irrational application of fertilizer can lead to negative economic and environmental consequences, such as high production costs, depletion of energy resources, and environmental pollution (GHG emissions and N leaching).

Nitrogen is a critical macroelement for plant growth and development, and its availability is the major limiting factor for primary productivity in most terrestrial ecosystems [9, 10]. In this sense, N fertilization is one of the most commonly widespread practices on agricultural productivity. Fertilizers, manure, N fixation by legumes and rainfall deposition are the main inputs of N to soils. According to the most recent FAO data [11], the consumption of N fertilizers in Brazil has increased by approximately 78% in the past 20 years. This over-application can result in inefficient use and high losses of N to the environment, which can impact air and water quality, biodiversity and human health [12]. For example, NO_3_^-^N is easily lost from agricultural areas by leaching [13]. This highly mobile form of N could lead to contamination of drinking water supplies and eutrophication of water bodies [14]. Furthermore, high levels of NO_3_^-^N can reduce nitrogen use efficiency (NUE) because the excessive N not taken up by plants is susceptible to loss [15, 16].

The Brazilian agriculture has achieved marked progress with regard to cereal production over the past two decades. Such an increase is the result, at least in part, of increased application of N fertilizers. Cereals such as rice (*Oryza sativa* L.), wheat (*Triticum* spp.) and corn (*Zea mays* L.) consume approximately 60% of the total N used as fertilizer and account for about one third of the total protein consumed worldwide [17]. The current world population of 7.2 billion is projected to increase by 1 billion over the next 12 years and to reach 9.6 billion by 2050 [18]. Therefore, recent projections have reported an estimated increase of 50 to 70% in cereal production from now to 2050 [6]. If there is not an increase in N use efficiency in coming years, the consumption of the N fertilizers will continue to increase. Thus, there is a need to identify strategies and agricultural practices to increase NUE.

The NUE for cereal production can be defined from the interplay of the following three different approaches: *agronomic efficiency*, which is generally defined as the grain yield per unit of applied N; *environmental efficiency*, which is characterized by the contamination of groundwater, surface water eutrophication, ozone depletion or greenhouse gas emissions caused by the release of N_2_O per unit of applied N; and *economic efficiency*, which is defined by the maximization of the farmer’s income per unit of applied N [19–21]. From an agronomic perspective, NUE is usually calculated using plot- or field-scale experiments [22–24]. However, in this study the NUE was thought as an agro-environmental indicator. This is a well-known approach and commonly used in the agro-policy context [25]. From this perspective, NUE can be calculated as the ratio between the amount of N removed by the crop and the amount of N fertilizer applied. This index provides information about the relative utilization of additional N applied to an agricultural production system in a country or region [26]. The calculated NUE for cereal production worldwide, using this agro-environmental index, is approximately 33% [20], which is far less than the 50% generally reported for crops [27, 28].

The export of N from the field due to crop harvest and N fertilization are the largest sources of output and input of N in an agricultural system. Therefore, the relationship between output and input of N can be used to describe NUE in agricultural production. In a theoretical system without any loss of N to the environment, an NUE of 100% would be ideal, as N input would correspond exactly to N output. Practically, this is not possible because agriculture operates in an open environment where there is continuous exchange of nutrients between the soil, environment, water and air [29].

Rational application of N fertilizer contributes to the efficient use of this nutrient in agricultural systems, but it does not necessarily optimize the NUE. Thus, a conflict occurs between desirable and acceptable levels of N fertilizer application in order to meet sustainable environmental standards. Farmers, extension workers, fertilizer industries, agricultural researchers and policymakers rarely take into account issues about how much N is being added to the system, what is maintained, and what happens to the excess N. Thus, the sustainable use of N fertilizer is critical not only to increase the N-use efficiency, but also to increase crop yield, reduce production costs, and improve environmental quality.

Here, we demonstrate the use of N fertilizers in the Brazilian agriculture by using the analysis of the seven major cereal crops (rice; wheat; corn; oats–*Avena sativa* L.; rye–*Secale cereale* L.; barley–*Hordeum vulgare* L.; and sorghum–*Sorghum bicolor* (L.) Moench). Our study reports on cereal production and N-use efficiency of N fertilizers in Brazil and their implications on GHG emissions. Studies with an emphasis on the assessment of N balance over a period of time in Brazil are missing. This study will help provide such information which will be useful for the design of public policies that deal with global environmental changes, especially in such a huge and diversified country like Brazil.

Practices seeking to increase NUE while decreasing N input in agricultural ecosystems are important not only to reduce environmental risks, but also to reduce the cost of agricultural production [30]. The objectives of this study were: (i) to analyze if application of N fertilizers results in an efficient use of N in cereal production in Brazil; (ii) to relate NUE with N_2_O emissions; and (iii) to estimate NUE and N_2_O emissions based on long-term N fertilizer use.

In a general view, our study aims to present a policy discussion about the N use in Brazil and related environmental problems (such as nitrous oxide emissions). According to Johnson et al. [31], in a macro environmental change context, it is very important to understand how increased application of N fertilizer affects GHG emissions.

## Materials and Methods

### Calculation of nitrogen-use efficiency (NUE)

The balance of N inputs/outputs in an agricultural system over time is a valuable parameter to understand the long-term fate of fertilizer-derived N in the plant-soil-water system and its impact on the environment. Nitrogen use efficiency (NUE) has been defined in several different ways in the literature and the methods to calculate it differ significantly [26, 32]. In plot- or field-scale experiments, NUE is commonly measured by establishing plots with and without applying N or with ^15^N labeled fertilizer [22–24].

From an agronomic perspective, both methods are quite accurate, although they usually show slightly different results. However, from a policy perspective, when NUE is considered to be an agro-environmental indicator, these methods are not feasible [25]. It is expensive and impractical to conduct extensive on-farm studies to determine NUE for several crops grown in different parts of the world. Therefore, in this study NUE was calculated by using an output/input ratio according to the methodology proposed by Raun and Johnson [20]:

$$NUE=\left[\left(N_{G}-N_{R}\right)/N_{C}\right].100$$ (1)

where *N*_*C*_ is the application of N fertilizer (in ton) for cereal production which corresponds to 53% of all N fertilizers for Latin America [33]; this value corresponds to an estimated amount of cereal N use calculated as weighted average of country-specific values [34]; weights were proportional to N use by countries. *N*_*G*_ is the cereal grain N removal calculated by multiplying N concentration in cereal crop by its yield; in this case, the values of N concentration (in g kg^-1^) for each crop are as follows: rice (12.3 g kg^-1^), oat (19.3 g kg^-1^), rye (22.1 g kg^-1^), barley (20.2 g kg^-1^), maize (12.6 g kg^-1^), sorghum (19.2 g kg^-1^), and wheat (21.3 g kg^-1^) according to Tkachuk [35]. *N*_*R*_ is the N removed by cereals coming from the soil natural fertilization (mineral N) or deposited by rainfall.

It is important to emphasize that the amount of N taken up in grain that comes from the non-N fertilizer sources (*N*_*R*_) varies due to several factors, such as N deposition rate, soil type, climatic conditions, and crop species. In field-controlled studies about a specific crop in a well-defined geographic region, it is possible to obtain a particular value of this parameter. Nevertheless, as our aim is to present a policy discussion about the N use in Brazil and some related environmental problems (such as nitrous oxide emissions) taking several cereal crops into account, it is impossible to calculate a precise value of *N*_*R*_. In such cases, there are different considerations in the literature. According to Freeman and Raun [36], *N*_*R*_ can range from 40 to 60% out of the amount of N taken up in grain. In addition, some authors [20, 37] have indicated that 50% of the N taken up in plants comes from mineralized N and atmospheric deposition. Finally, ^15^N labeled fertilizer experiments show that most part of the N taken up in plants comes from the soil (50% or more), even in well-fertilized crops [38, 39]. Thus, faced with these evidences, *N*_*R*_ = *N*_*G*_ x 0.5 has been chosen to be considered in this study. By recognizing that there is some degree of uncertainty in *N*_*R*_, a confidence interval for each NUE value calculated was added based on the variability of N use in Brazil (*N*_*C*_).

The index (Eq 1) was calculated for Brazil between 1970 and 2011. Furthermore, NUE was estimated for the coming years (2015 to 2023) by using the long-term N fertilizer demand data (see calculation of long-term N fertilizer demand for cereal production). It was assumed that the main cereal crops in Brazilian agricultural production are rice, oats, rye, barley, corn, sorghum and wheat. Although legumes such as beans and soybeans are very N demanding, they were left out of the analysis because biological inoculant is used for N fixation without supplying N fertilizers.

According to Brentrup and Palliere [25], the agro-environmental indicator (Eq 1) provides evidence “about the relative utilization of additional N applied to an agricultural production system of a country or region”. By using this indicator, it is possible to evaluate advances or to compare different regions over time.

### Calculation of GHG emissions from cereal production

To estimate GHG emissions related to N fertilization in cereal production, we adopted the methodology proposed by De Klein et al. [40] and Crutzen et al. [41] with some modification. The N_2_O is produced naturally in soils through the processes of nitrification and denitrification. One of the main controlling factors in this reaction is the presence of inorganic N in the soil. This methodology estimates N_2_O emissions through N fertilization (e.g., synthetic or organic fertilizers). The emissions of N_2_O occur through both direct (nitrification and denitrification with N input) and indirect (NH_3_ volatilization, N leaching, and surface runoff) pathways.

The N_2_O emissions from applied N fertilizer were calculated for Brazil between 1970 and 2011. In addition, N_2_O emissions were estimated for the coming years (2015 to 2023) using the calculated long-term N fertilizer demand data (see calculation of long-term N fertilizer demand for cereal production). According to De Klein et al. [40], direct N_2_O emissions from N fertilizer constitute 1% of the total applied N fertilizer. Similarly, indirect N_2_O emissions due to NH_3_ volatilization, N leaching, urea hydrolysis, and surface runoff constitute about 0.4% of N fertilizer applied [41]. In this way, direct and indirect N_2_O emissions from N inputs were calculated according to the following equations:

$$N_{2}O_{Ninputs}=N_{2}O_{direct_{Ninputs}}+N_{2}O_{indirect_{Ninputs}}$$ (2)

$$N_{2}O_{{direct}_{Ninputs}}={FS}_{N}.{EF}_{1}.310$$ (2a)

$$N_{2}O_{{indirect}_{Ninputs}}={FS}_{N}.{EF}_{2}.310$$ (2b)

where *FS*_*N*_ is the amount of N synthetic fertilizer applied to soils (kg N yr^-1^); *EF*_*1*_ is the emission factor for direct N_2_O emissions from N inputs (kg N_2_O-N kg N input^-1^) or 0.01 [40]; *EF*_*2*_ is the emission factor for indirect N_2_O emissions from N inputs (kg N_2_O-N kg N input^-1^) or 0.004 [41]; and 310 is a dimensionless factor to convert N_2_O to CO_2_ eq., i.e., the global warming potential (GWP) for N_2_O over a 100-year time horizon [42].

### Calculation of long-term N fertilizer demand for cereal production

The growing world population will likely require more intensive agricultural crop production. Higher productivity will, in turn, increase the demand for agricultural inputs, such as N fertilizers [43]. We calculated the amount of N fertilizer needed to support the FAO-OECD projections for cereal production in Brazil from 2014 to 2023 according to a causal model proposed by Tenkorang and Lowenberg-DeBoer [8].

Causal models are useful when projections of the exogenous variables are available, such as production and cultivated area [44]. Previous models have demonstrated the correlation between future fertilizer requirement and past fertilizer status as well as past and future crop production based on agronomic relationships as follows [8, 43]:

$$F_{t}=F_{t-1}+\left(Y_{t}-Y_{t-1}\right)/\left(Y_{t-1}/F_{t-1}\right)$$ (3)

where *F*_*t*_ is the unadjusted N fertilizer application rate in year *t*; *F*_*t-1*_ is the unadjusted N fertilizer application rate in year *t – 1*; *Y*_*t*_ is the crop yield in year *t*; and *Y*_*t-1*_ is the crop yield in year *t – 1*. According to FAO [43], “in the absence of known crop production functions, future fertilizer application rates and nutrient use efficiencies were estimated by first quantifying the relationship between production and total fertilizer application rates”. Following the studies of FAO [43] and Tenkorang and Lowenberg-DeBoer [8], we predicted that (1) the relationship between the actual N fertilizer use and the previous year’s N fertilizer use as well as expected crop production would be positive and (2) the relationship between the actual N fertilizer use and the previous year’s crop production would be negative. Relationship (1) suggests that strong N fertilizer-consuming countries may continue to consume more and use rates in increasing crop yields over time. Relationship (2) suggests that ‘a good year is followed by a bad year’ crop yield in certain poor areas.

Due to availability of historical, current and projected data, the following N fertilizer demand model was adopted:

$$FN_{t}=\alpha_{0}+\beta_{1}Y_{t}+\beta_{2}Y_{t-1}+\beta_{3}FN_{t-1}+\varepsilon_{t}$$ (4)

where *FN*_*t*_ is the amount of N fertilizers used in year *t*; *Y*_*t*_ is total cereal crop yield in year *t*; *Y*_*t-1*_ is the total cereal crop yield in year *t – 1*; *FN*_*t-1*_ is the amount of N fertilizers used in year *t – 1*; the coefficients *β*_*1*_, *β*_*2*_ and *β*_*3*_ are the unknown parameters which describe the dependence of *FN*_*t*_ on *Y*_*t*_, *Y*_*t-1*_ and *FN*_*t-1*_; *α*_*0*_ is the intercept parameter (the value of *FN*_*t*_ when each of the explanatory variables takes the value zero); and *ε*_*t*_ is the random error term.

Our analysis considered the period between 1970 and 2011. However, similar to the model proposed by Tenkorang and Lowenberg-DeBoer [8], the last three years (2009–2011) were arranged as out-of-sample data in order to estimate the model forecasting power. Thus, the predicted model for the 1970–2008 period was used to estimate N fertilizer amounts for the years 2009, 2010 and 2011. These estimated amounts were compared to their actual values using mean absolute percentage error (MAPE):

$$MAPE=\frac{1}{n}\sum_{t=1}^{n}\left|\frac{A_{t}-F_{t}}{A_{t}}\right|$$ (5)

where *A*_*t*_ is the actual value; *F*_*t*_ is the estimated N fertilizer amounts; and *n = 1*, *2* and *3* (2009–2011).

Finally, the estimated model was used to forecast N fertilizer demands from 2014 to 2023 based on the projected cereal output.

### Calculation of N fertilizer savings

After calculating NUE, the potential N fertilizer savings (ton) per year for each percentage of increase in NUE under current fertilizer use and agricultural production was estimated [20]. To establish this increase percentage, the geometric growth rate (GGR) of NUE in the 41 years (1970–2011) was estimated as:

NUE=α+βt+ε (6)

where *t* is the trend variable; *β* is the unknown parameter that describes the dependence of *NUE* on trend variable (*t*); *α* is the intercept parameter (the value of *NUE* when *t* takes the value zero); *ε* is the random error term; and *GGR* = [*anti* log(*β*)−1].100.

Once GGR was defined, the NUE formula (Eq 1) was used in order to answer this question: how much N (ton) is necessary so that the current value of o NUE grows as much as GGR? This N value obtained is lower than the previous one because the efficiency increased. The difference between both values of N (the actual one and the one resulting from the simulated increase of NUE) represents the fertilizer savings (ton). Similar to Raun and Johnson [20], we also did this calculation considering 1% increase in NUE. Using these values of the potential N fertilizer savings (ton), the value of N savings in US$ was calculated based on the average price of three N fertilizers (i.e., urea, ammonium nitrate and ammonium sulfate) in 2014.

### Data analysis

The Brazilian annual total consumption of N fertilizer (in ton), cereal production (for rice, oats, rye, barley, corn, sorghum and wheat, in ton), and cultivated land under cereals (in ha) from 1970 to 2011 were obtained from the FAOSTAT [11] website (see S1 and S2 Tables for a detailed overview of cereal production in Brazil). Furthermore, the projected data of Brazilian cereal yields and cultivated land for cereals from 2014 to 2023 was obtained from the OECD-FAO Agricultural Outlook database. The average price of N fertilizers (urea, ammonium nitrate and ammonium sulfate) for the nine major cereal-producing states in Brazil (i.e., Rio Grande do Sul, Paraná, Mato Grosso, Goiás, Santa Catarina, Mato Grosso do Sul, São Paulo and Minas Gerais) in 2014 was obtained from the Companhia Nacional de Abastecimento (CONAB). The structural-causal model described in the section Calculation of long-term N fertilizer demand for cereal production (Eq 4) and geometric growth rate (Eq 5), were estimated using ordinary least squares (OLS). The OLS estimates and the model-data fit (R^2^, Coefficient of Variation, ANOVA, and tests of Multicollinearity, Autocorrelation and Heteroskedasticity) were obtained using Stata 12 software (StataCorp LP, College Station, TX, USA).

## Results and Discussion

### Current and historical NUE trends

In Brazil, the consumption of N fertilizer for cereal production increased by 78% in the past 20 years. In contrast, the area where the major cereals are cultivated in Brazil did not change significantly during the same time period. Accordingly, the use of N fertilizers per unit area increased (S1 Table), which suggests a greater agricultural intensification considered as an increase in the amount of external inputs (N fertilizers). The expansion of agricultural land was neglected in the analysis of cereal production at least in the period of time assessed. However, since agricultural intensification also comes at a cost to the environment, our study aimed at evaluating whether such intensification led to efficiency or not, and also the consequences of the possible lack of efficiency.

**Fig 1:**
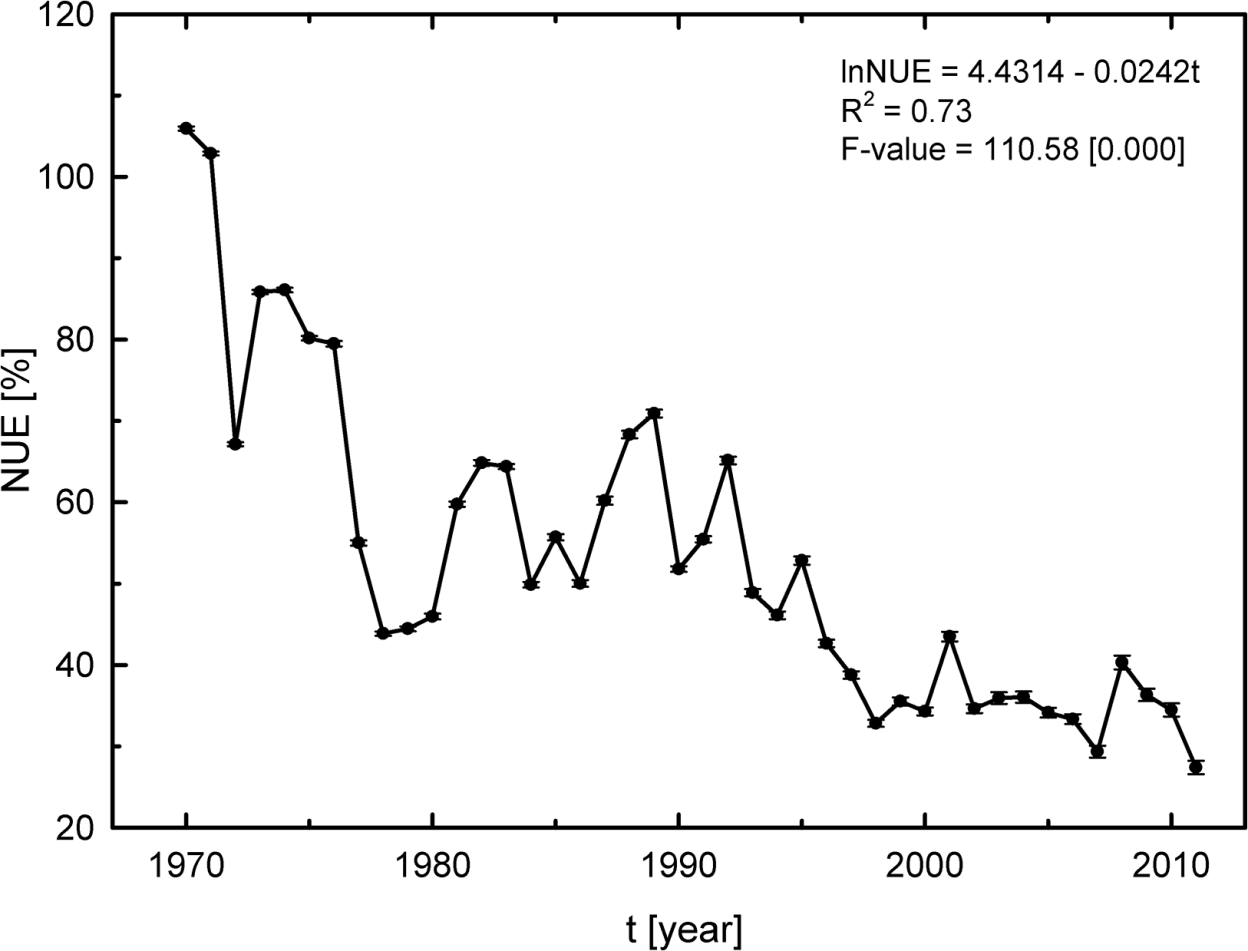
Calculated nitrogen use efficiency (NUE) for Brazil, 1970–2011. Data are shown for the seven major cereal crops (rice, oats, rye, barley, corn, sorghum, and wheat). NUE data are based on the author’s calculation from the estimated model (Eq 1). The geometric growth rate (GGR) was estimated from GGR = [*anti*log (β) – 1].100 where the parameter *β* was estimated from the Eq 6. All coefficients were statistically significant at 1%. See S4 Table for additional details of ANOVA and Model fit.

First, the NUE for cereal production between 1970 and 2011 was calculated using an output/input ratio (Fig 1). An approximate 74% decrease in the NUE was observed with a 2.39% decreasing rate per year. Although no economic loss was recorded (since the agricultural productivity increased at the same time, as can be observed in S1 Table), it is noteworthy that the increasing use of N fertilizers did not lead to efficiency. It is likely that such inefficient use of N fertilizers is related to environmental losses, mainly due to the open nature of an agricultural system [29].

The world cereal-grain NUE is estimated to be 33% [20]. The same authors reported a cereal NUE of 29% in developing countries. Likewise, the cereal NUE calculated for Brazil in 2011 was 27% (Fig 1) while the remainder pollutes the air and the ecosystems, which indicates that the country is below the NUE limit values for cereal production systems. Denitrification, volatilization and leaching are among the possible causes for the low NUE values found for Brazil, and these issues usually reflect irrational and unsustainable agricultural practices. Studies have reported that cereal plants release N, mainly as NH_3_, from their tissues after anthesis [45–47]. Additionally, when N fertilizers are applied at rates higher than those required for maximum yields in cereal crops, NO_3_^-^ leaching can be significant [20, 23].

In field studies with coffee in Brazil, average ^15^N fertilizer recovery at harvest was 34% in the first cropping year and 47% in the second year [48]. Moreover, total ^15^N fertilizer recovery was 36% for the 3-yr-old ‘Valencia’ orange trees and 52% for the ‘Lisbon’ lemon trees [49]. In addition, the average amount of ^15^N fertilizer recovered by sugarcane under Brazilian field conditions was 20% near the harvesting stage [50]. The remainder is stored in soil organic matter pools, immobilized in the soil or lost from the cropping system. Taken together, these results emphasized a low N recovery in Brazilian field conditions compared to crops other than cereals.

Another relevant factor with regard to low NUE is the complacency of farmers. Depending on the source of fertilizer used, N costs are approximately $ 0.42 kg^-1^. This affordable price compared to the Brazilian farmers’ per capita income (~$ 12,500.00) is therefore likely to explain the over-application of N fertilizer. Moreover, high efficiency should not be prioritized at the expense of productivity. According to the law of diminishing returns, increases in productivity tend to be smaller with the increase of N amounts resulting in lower efficiency gains [51].

Finally, it is important to emphasize that, based on current fertilizer use and production rates, a 1% increase in NUE for cereal production would save 21419.86 ton in N fertilizer use, accounting for $ 9 million savings in N fertilizer costs. Furthermore, assuming the estimated geometric rate (NUE decrease rate over the period 1970–2011), a 2.39% increase in NUE would save 50531.25 ton in N fertilizer, accounting for $ 21 million savings in N fertilizer expenditure. In comparison, Raun and Johnson [20] reported that a 1% increase in the efficiency of N use for grain production worldwide would lead to $ 234 million savings. These results combined indicated that increases in NUE rates would lead not only to agronomic and environmental benefits but also to economic improvement for farmers.

### GHG emissions related to N fertilization from cereal production in Brazil

**Fig 2:**
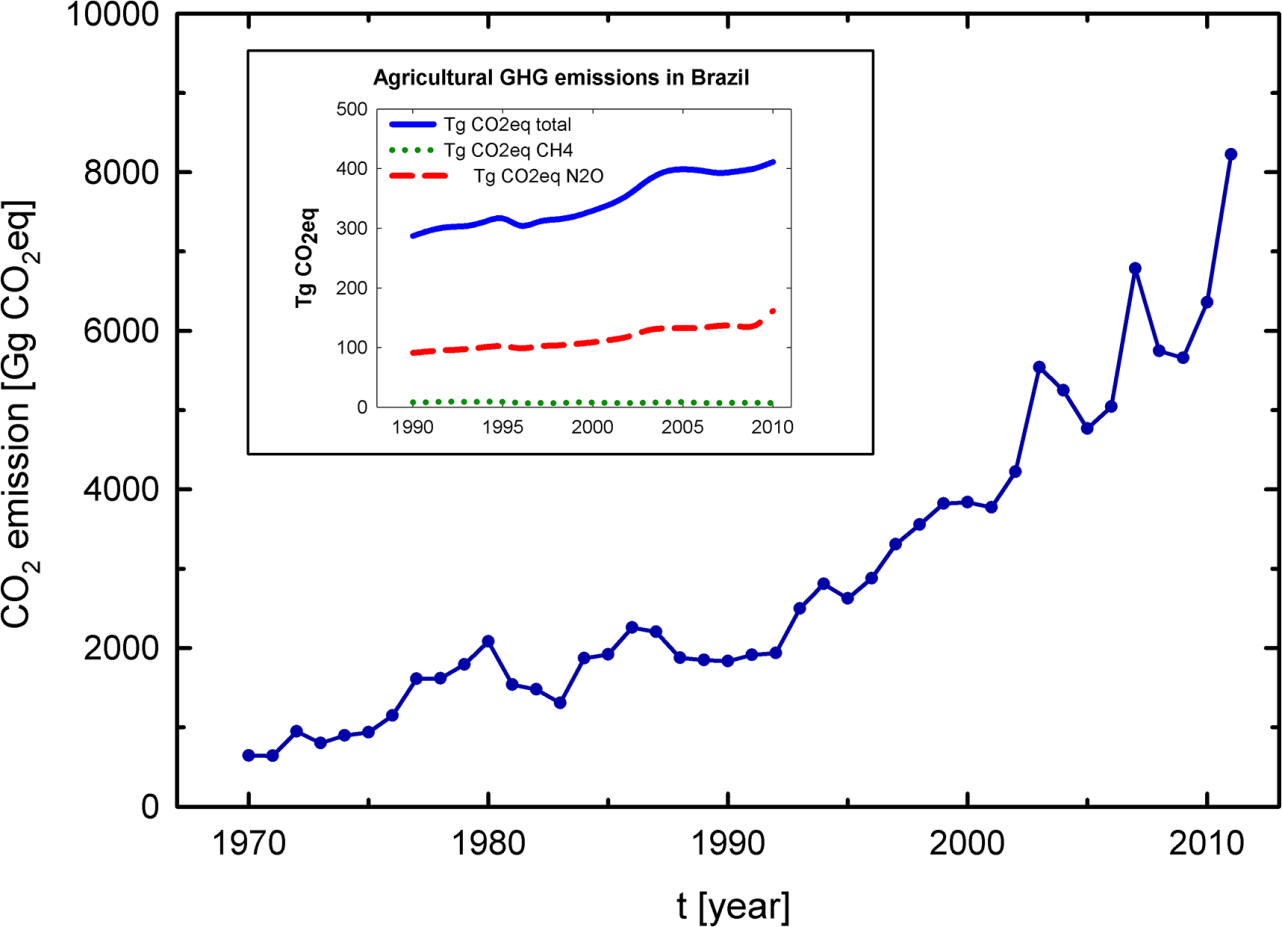
CO_2_ eq. emission trends related to nitrogen fertilization from cereal production for Brazil, 1970–2011. CO_2_ eq. emissions (1Gg = 1000 ton) from N fertilization data are based on the author’s calculation from the estimated model (Eq 2). The inset graph illustrates the agricultural GHG emissions in Brazil from 1990 to 2010 according to MCTI report [4].

Based on the premise that agriculture is a major source of GHG emissions, we estimated the GHG emissions related to N fertilization from cereal production in Brazil from 1970 to 2011. Fig 2 shows a dramatic increase in CO_2_ eq. emissions from cereal crops by approximately 12 times between 1970 and 2011. The agricultural sector is dominated by CH_4_ emissions from livestock due to enteric fermentation as well as manure. Furthermore, N_2_O direct (from grazed grassland) and indirect (by N atmospheric deposition and leaching) emissions are responsible for a large amount of total agricultural GHG emissions [3]. Atmospheric emissions may occur as nitrogen dioxide (NO_2_), nitric oxide (NO), nitrogen gas (N_2_), N_2_O or NH_3_, and water bodies receive NO_3_^-^ and dissolved organic N inputs by leaching and runoff [52]. Moreover, the manufacturing processes of N fertilizers are responsible for part of the direct emissions of CO_2_ to the atmosphere [29].

N_2_O is not a reactive gas in the troposphere, but it is a potent non-CO_2_ GHG. On a molar basis, N_2_O is approximately 300 times more potent than CO_2_ [42]. The atmospheric concentration of N_2_O has increased by nearly 18% since the pre-industrial period [53]. Such increase has contributed to about 6% of total GHGs that drive climate change [42]. Moreover, around 80% of the N_2_O added to the atmosphere annually by human activities is derived from agriculture. More specifically, approximately 60, 30 and 10% of these emissions come from agricultural land, animal waste management, and burning agricultural green waste, respectively [53, 54]. Thus, N_2_O is an important target for compensation projects, which may be included in *cap and trade* markets due to high returns associated with N_2_O emission mitigation [29].

Several field experiments have shown that the amount of N fertilizer applied is the strongest indicator of N_2_O fluxes in major cropping systems [55–59]. In addition to the amount of N applied, the N_2_O flow can also be influenced by the N fertilizer formulation and application timing as well as by the agronomic practices that determine N availability in the soil, such as tillage and waste management [29]. Recent studies have reported that approximately 0.5–3% of N applied to cultivated soils is emitted as N_2_O to the atmosphere [56, 59]. Nevertheless, the emission rates can be even higher if the level of N fertilization exceeds crop demand [55, 57, 58].

Fig 2 shows that the emission related to N fertilization from cereal production reached an amount of nearly 6.5 million ton of CO_2_ eq. in 2010. In comparison, agricultural and livestock activities were responsible for the emission of 437 million ton of CO_2_ eq. in the same year in Brazil, and N_2_O contributed to 161 million ton of CO_2_ eq., i.e., which is approximately 37% of total emissions from the agricultural sector [4]. Moreover, the application of synthetic fertilizer was responsible for both direct and indirect (atmospheric deposition + leaching) emissions of 24 million ton of CO_2_ eq. in 2010 in Brazil [4]. Such results suggest that the emission derived from N fertilization for cereal production represents 27% of the total synthetic fertilization emission contribution from the Brazilian agricultural sector.

### Projections of long-term N fertilizer demand highlight the need to improve NUE

In order to obtain the long-term N fertilizer requirement forecasts, the structural-causal model described in Eq 4 was estimated. The model R^2^ was above 0.9, and the *F*-statistic was highly significant (P-level: <10^−3^). The CV in the structural model was 17.25, which suggests a good model fit. Only the coefficient of the lagged crop output (*Y*_*t–1*_) was not significantly different from zero, and the other coefficients were statistically significant at the 1% or 5% test level when considering the White robust standard error terms. We also tested the model for the presence of multicollinearity by using the variance inflation factor (VIF). According to the results, the VIF values ranged between 10.9 and 15.5. Although VIF values lower than ten are desirable, O’Brien [60] stated that a VIF up to 40 can be tolerated and does not undermine the regression analysis. It is important to highlight that there was no autocorrelation in the model. The Durbin Watson statistic test (Durbin’s *h* test) was not statistically significant. Table 1 shows a detailed overview of the model estimation.

**Table 1:** Nitrogen fertilizer forecast model estimates for cereal production in Brazil.

	*Coefficient*	*Bootstrap Std*. *Error*	*P-value*	*VIF*
Crop Output (*Y*_*t*_)	0.0100^**^	0.0040	0.0130	11.95
Lagged Crop Output (*Y*_*t-1*_)	-0.0022	0.0043	0.6020	10.93
Lagged N Fertilizers (*FN*_*t-1*_)	0.7486^***^	0.1949	0.0000	15.51
Constant	-141224^*^	80129.6	0.0780	—-
*Model fit*				
*R* ^*2*^: 0.9342; Model *CV*: 17.25				
*F-value*: 175.16 [0.0000]				
*Durbin stat (DWh)*: 0.468 [0.4937]				

Sample size, N = 41. Asterisks indicate statistically significant differences at 1% (***), 5% (**), and 10% test level (*) considering the White robust standard error terms (estimated by bootstrap – 1000 replicates). The data in brackets for the model fit are P-values. See S5 Table for additional details of ANOVA.

**Fig 3:**
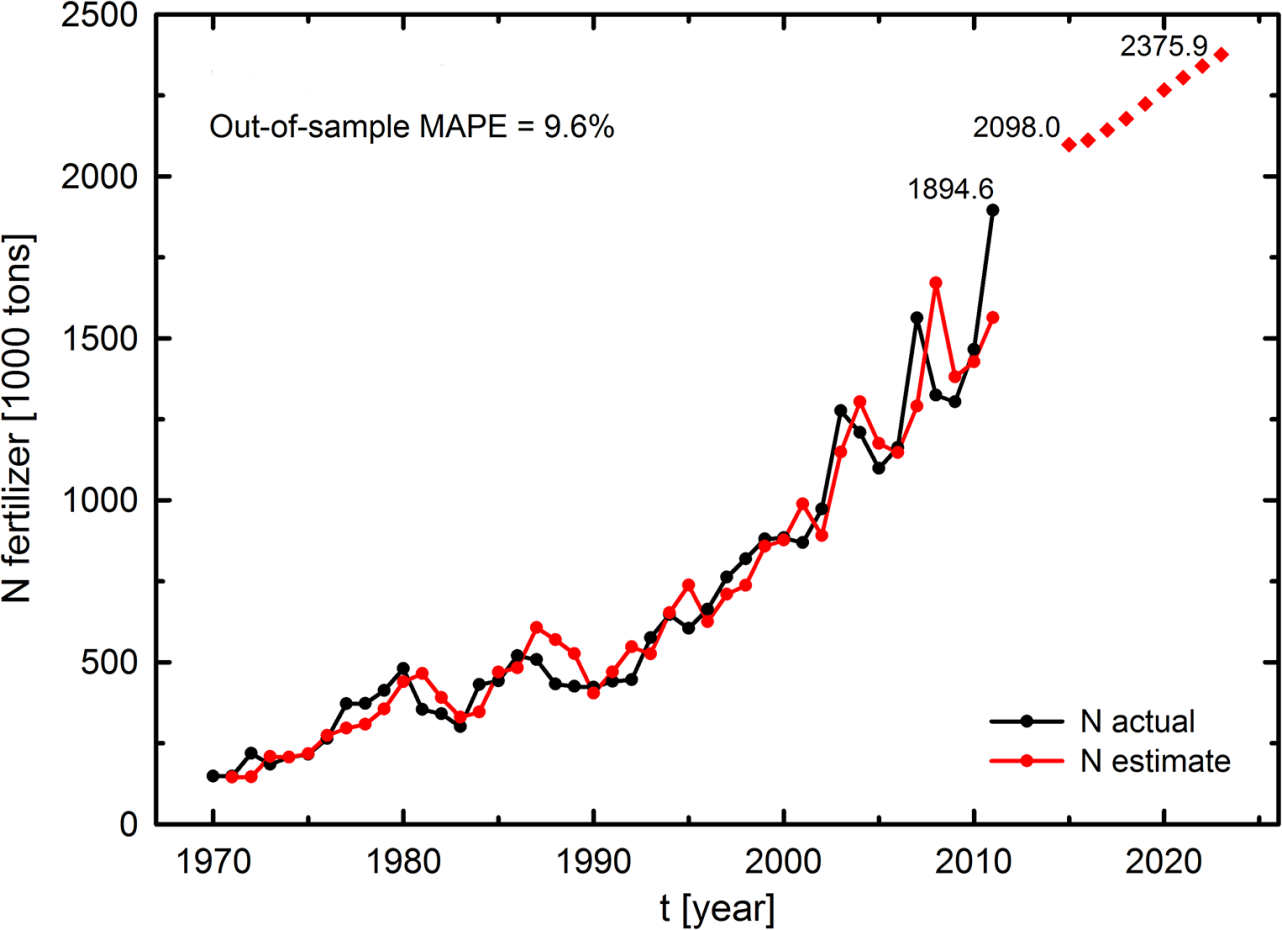
Long-term nitrogen fertilizer demand for cereal production in Brazil. Actual versus estimate forecast (1970–2011), and nitrogen fertilizer requirement forecasts for 2015 to 2023 were calculated using the estimated model (Eq 4).

The model forecasting power was also validated by using the mean absolute percentage error (MAPE). When comparing the actual N fertilizer consumption and the model-estimated consumption, the models tracked the historical data effectively (Fig 3). The MAPE for the out-of-sample forecasts (2009–2011) was 9.6%, which is an acceptable value [8].

The estimated causal model offered additional evidence in relation to the inefficiency of N use in Brazil and corroborated with the above-mentioned results (Fig 1). This evidence resulted from evaluating the elasticity of fertilizer use with respect to output (evaluated from the average output and average fertilizer use) calculated by the product of the output coefficient (*β*_*1*_ in the Eq 4) and the ratio of average crop output (*Y*_*t*_) to average fertilizer use (*FN*_*t*_). The value indicates the percentage change in *FN*_*t*_ due to a 1% change in *Y*_*t*_. Elasticity estimated for the 1970–2011 time period was 0.65. According to Tenkorang and Lowenberg-DeBoer [8], elasticity less than one are a good indication of nutrient drawdown, i.e., inadequate N application, indicating soil nutrient depletion. A long-term drawdown is likely to increase environmental and agricultural risks associated with low NUE and soil moisture as well as to decrease farm income capacity [61].

Using the causal model, N fertilizer forecasts for cereal production in Brazil were projected. The estimated values were 2.09 million ton and 2.37 million ton for 2015 and 2023, respectively, at a growth rate of 1.67% (Fig 3). Several recent reports have suggested that the increases in the projected crop production during the next 30 years are likely to require the increased use of fertilizers, particularly N [8, 62, 63]. Based on an econometric model, Bumb and Baanante [62] estimated that the world fertilizer demand will increase by 1.2% annually from 1992 to 2020. Dyson [63] estimated that the world use of mineral N will double by 2025 at a growth rate of 2.26%. According to Tenkorang and Lowenberg-DeBoer [8], the projected global N fertilizer forecasts are 115 million ton and 137 million ton for 2015 and 2030, respectively. The same authors reported that Latin America will consume approximately 5.3 million ton of N fertilizer in 2015 [8]. In comparison, the projected N fertilizer consumption for Brazil will account for 40% of the total Latin American consumption in 2015.

It is noteworthy that our baseline growth rate for the N fertilizer demand between 1970 and 2011 was 5.41% per year, and the projected growth rate (from 2015 to 2023) will decrease to 1.67%. Thus, how this trend would lead to NUE and to possible decreases in GHG emission rates was also investigated. Intriguingly, a sharp increase in the projected NUE rates was observed (S3 Table). Based on the model estimated by Eq 4 and its related parameters shown in Table 1, NUE is expected to increase 0.02% annually from 2015 to 2023. In contrast, a decrease rate of 2.39% per year was observed between 1970 and 2011. Thus, such results highlight an optimistic scenario for cereal production in Brazil related to the efficiency of N use in coming years. This change in the NUE trend is clearly related to the lower growth rate for N fertilizer projected by our model. In addition, this is a simulation of a more optimistic scenario, in which sustainable agricultural practices with regard to the use of N are expected to increase. However, some caution is necessary, and a more detailed dialogue should be encouraged.

Given its importance in the maintenance of soil fertility, N has been intensively studied in order to improve NUE from an agronomic, physiological and genetic perspective. These experimental efforts aim at reducing N loss from the soil and at improving the mechanism of absorption and N metabolism within the plant. However, studies emphasizing the *trade-off* between economic development and the impact of the use of agricultural inputs on GHG emission are scarce. Thus, it is of pivotal importance that economic policies include environmental issues in their agenda due to the relevance of such issues to agricultural productivity and food security.

More efficient use of N through improved timing, split applications, site-specific management, crop rotation, crop diversification, soil testing, biological N fixation (BNF) and improved plant traits by genetic breeding can help improve yields with the same amount or even a smaller amount of N fertilizers. Biological nitrification inhibition (BNI) by *Brachiaria* roots exudates represents another important strategy to improve NUE in Brazilian soils [64, 65]. The process of nitrification (i.e., the biological oxidation of NH_4_^+^ in NO_3_^-^ by bacteria of the genera *Nitrosomonas* and *Nitrobacter*) increases N leaching and atmospheric losses. It is interesting that most of the Brazilian soils are well-drained and present neutral to slightly acidic reaction characteristics that favor the prevalence of N in the NO_3_^-^ form [66]. In this sense, nitrification inhibitors are widely used and can help reduce losses of N in soil that would otherwise be used by crops. These characteristics reinforce the relevance of using *Brachiaria* as a BNI tool in Brazilian soils, especially in crop rotation strategies.

In this sense, the adoption of agricultural practices that enable increased NUE to replace conventional practices should be highly encouraged. For example, for an improved NUE, farmers can reduce N fertilization to levels that still provide satisfactory yields [67]. In addition, there is a lag between the release of N applied and its uptake by the plants. In general, after the application of N to soil, its availability decreases over time, but the crop requirement increases. Thus, N fertilizer applied at the correct time maximizes the effect of N and minimizes potential environmental losses [29].

Additionally, the development of cultivars with high agronomic NUE is an economically viable option to ensure higher productivity in agricultural systems with a low use of inputs [68–70]. Thus, further efforts are needed to enhance the selection of plants with high rates of NUE, which is not often seen as a priority by plant breeders [20]. Ongoing investment in plant breeding research and development is vital to ensure continued advances in order to obtain cultivars that efficiently use the available N sources and reduce farm costs and associated losses.

Several N management practices to improve NUE of agricultural systems are already available and only require appropriate support to be adopted. Such strategies should be prioritized, and more rational methods of N fertilizer application should be developed to avoid excessive fertilization, including the following practices: *i*) fertilizer placement because it affects its availability for crop uptake as well as its susceptibility to soil transformations; *ii*) rate or amount of N fertilizer applied because this affects the amount of GHGs emitted more than any other factor; *iii*) fertilizer timing, which represents a major challenge for efficient fertilizer management and aims at synchronizing soil N availability with crop N demand; and *iv*) N fertilizer formulation and additives [29]. For instance, controlled release fertilizers (CRFs) enhance the efficiency of N uptake and minimize losses to the environment, and they represent a feasible alternative to conventional practices. However, the acceptance of CRFs by farmers remains limited worldwide due to lack of experience with the technique and its high relative cost [71, 72].

Generally, any practice that increases NUE is expected to reduce GHG emissions because the N absorbed by the plant is not available to soil processes that drive N emissions at least in the short term [29]. For example, good N management practices, such as crop rotation, have been shown to decrease N_2_O emissions [73, 74]. However, decreases in our projected CO_2_ eq. emissions were not observed alongside NUE increases. We estimated a value of CO_2_ eq. emission 24% higher in 2023 (~10.5 million ton of CO_2_ eq.) in comparison to 2011 (~8 million ton of CO_2_ eq.). Our results were in agreement that practices improving NUE do not always reduce GHG emissions [29]. For instance, different N fertilizer formulations or additives can result in different N_2_O emissions despite presumed NUE effects. In addition, banded fertilizer placement can increase NUE but also increase N_2_O emissions. In general, NUE is an important parameter, but by itself it is not enough to decrease GHG emissions [29].

In this sense, specific policies that stimulate sustainable agriculture practices mitigating GHG emissions are needed. As the world population grows and the demand for agricultural commodities increases, sectorial plans to mitigate GHG emissions can contribute significantly to achieve the international goals for the reduction of future global emissions and the stabilization of atmospheric concentrations within safe levels [75].

Brazil has become an international example regarding mitigation plans once it voluntarily committed to reducing its GHG emissions in 2009. The National Climate Change Plan (NCCP) has the goal of having reduced GHG emissions by 36.1% to 38.9% by 2020 considering a baseline scenario. The estimated reduction is approximately one billion ton of CO_2_ eq. [76]. In the context of the Brazilian NCCP, the so-called Low-Carbon Agriculture (ABC) program provides resources and incentives for farmers who adopt sustainable agricultural practices (e.g., no-till systems, degraded pastureland rehabilitation, integrated crop-livestock-forestry systems, planted forests, BNF, and animal waste management).

Our future projections of NUE and N fertilizer demand (Fig 3) are likely to indicate long-term benefits to the Brazilian NCCP. Increases when adopting agricultural practices such as no-till systems or crop rotation/diversification should contribute to decreases in N fertilizer demand growth rate and to increases in NUE in following years. No-till cultivation improves soil structure and enhances the nutrient retention of plants [77]. When we take into consideration the Brazilian agricultural production as a whole, N input through BNF (a microbiological process that converts atmospheric N into a plant-usable form) can help maintain soil N reserves and substitute for N fertilizer to attain large crop yields [78, 79]. However, Brazilian farmers remain skeptical about the ABC program being able to reduce agricultural carbon emissions [80]. In this context, it is of great importance that the ABC program be strictly enforced [81].

**Fig 4:**
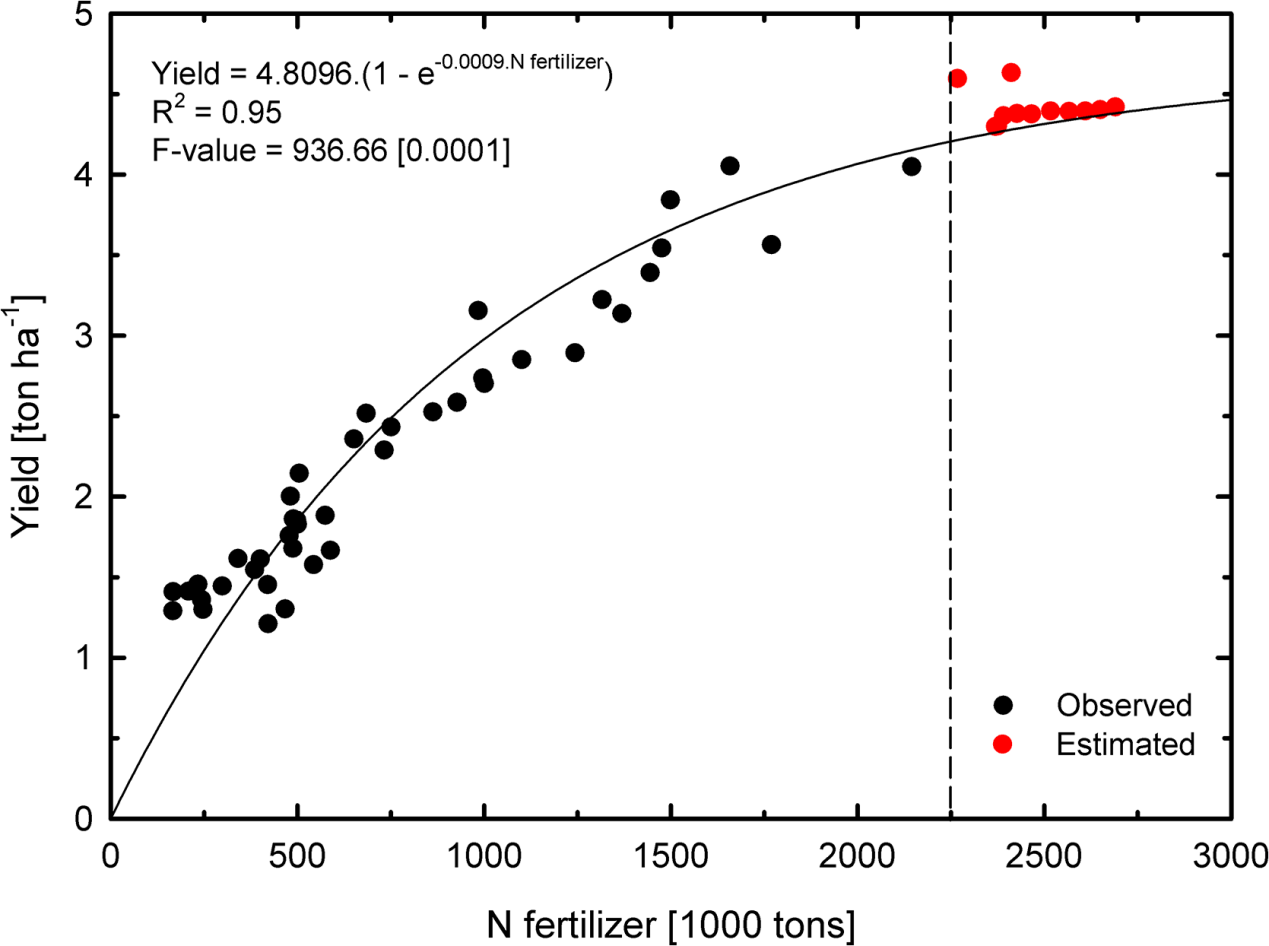
Effect of nitrogen fertilizer consumption on cereal yield for Brazil, 1970–2023. Observed (1970–2011) and estimated (2015–2023) relationships between N fertilization and yield. The trend line represents an exponential model fit. All coefficients were statistically significant at 1%. See S6 Table for additional details of ANOVA and Model fit.

Although some optimism can be encouraged from our forecasting results, it should highlight that Brazil has not yet reached a point at which it is possible to reduce the use of N fertilizers without compromising cereal yields. More importantly, a continuous increase in temporal yield in relation to N fertilizer use was found, and those increases are likely to persist in estimated forecasts (Fig 4). A typical N response curve based on field or greenhouse studies shows that applying N gives a large increase in yield but applying too much of it can reduce yield due to salinity damages, specific nutrient toxicities, and other factors [82–84]. However, this does not happen on a regular basis at the farm because farmers do not typically apply those high rates, unless it is a mistake. Finally, our results emphasized a country-level temporal trajectory of a high environmental footprint and also the need for specific policies aimed at reducing the unsustainable N fertilizer consumption.

One of the main challenges of modern agriculture is coping with the increasing world population on one hand and the magnitude of food production on the other hand. Some recent works point to Brazil as one of the main food suppliers worldwide in the coming years [82]. In this sense, the relationship between cereal yields and N fertilization is not only of interest to understand NUE, but also helps stimulate and formulate policies to increase NUE and reduce N input into the agricultural systems [83, 84]. In addition to those already-mentioned strategies to improve agronomic practices [29, 71, 72, 82], an increased NUE can be obtained by demand-side mitigation measures, such as a reduction in household waste and less consumption of livestock-based products [83]. Furthermore, it is noteworthy that economic instruments like taxes on N fertilizers should be associated with investments in Research and Development that effectively stimulate improvements in agronomic techniques to ensure a rational N use.

## Conclusions

Our study aimed at investigating the relationship between the evolution in cereal production (rice, oats, rye, barley, corn, sorghum and wheat) and the use of N fertilizers in Brazil concerning the efficiency of these fertilizers and their implications for producing GHG emissions. Evaluations included the N use efficiency (NUE), the contribution of N fertilization to GHG emissions, and the long-term N fertilizer demand for cereal production in Brazil. In addition, projections of the association between NUE and GHG emissions were made.

A decrease in NUE of approximately 74% was observed between 1970 and 2011. Therefore, the inefficient use of N fertilizers is directly related to environmental losses, such as GHG emissions, mainly N_2_O. Our results suggest that the emissions derived from N fertilization in cereal production represent 27% of the total synthetic fertilization emission contribution from the Brazilian agricultural sector in 2010. Based on current fertilizer use and production rates, a 2.39% hypothetical increase in NUE would lead to 50531 ton savings in N fertilizer, which accounts for $ 21 million savings in N fertilizer expenditure.

Interestingly, low values of NUE do not mean that all N not in grain is lost. Fertilization of lower quality soils, along with the increased addition of organic matter, can result in increased soil organic carbon and nitrogen. This process is not likely to be responsible for retaining most of the unaccounted N, but it should not be ignored in our analysis. Additional studies are required to clarify the importance of NUE in lower quality soils.

Based on the predicted scenario of the increase in N fertilizer consumption in coming years, our results indicated that increases in NUE rates would lead not only to agronomic and environmental benefits but also to economic improvement. However, these improvements can only be achieved by adopting sustainable practices that help to maximize NUE and encourage the rational use of agricultural inputs.

In summary, these are our suggestions for a pathway to NUE improvement in Brazil: *i*) the adoption of agricultural practices that enable increased NUE (e.g., crop rotation, no-till systems, BNI and BNF) to replace conventional unsustainable agricultural practices; *ii*) the development of cultivars with high agronomic NUE through genetic breeding, which efficiently utilize available N sources and consequently reduce farm costs and associated losses; *iii*) the use of more rational methods of N fertilizer application in the field to avoid excessive fertilization; and *iv)* the implementation of demand-side measures aimed at reducing household waste and animal products consumption. Some of these opportunities are currently available, but many others require more empirical and theoretical research to be confirmed as potential strategies. All such opportunities require effective incentives to actually become a reality. Thus, it is of pivotal importance that economic policies include environmental issues in their agenda based upon the relevance of such issues in agricultural productivity and food security.

## Supporting Information

S1 Table Overview of N fertilizer consumption and associated cereals production in Brazil from 1970 to 2011. (DOCX)

S2 Table Overview of the seven major cereal crops (rice, oats, rye, barley, corn, sorghum, and wheat) production and cultivated area in Brazil from 1970 to 2011. (DOCX)

S3 Table Nitrogen use efficiency (NUE) and CO_2_eq emission related to N fertilization from cereal production forecast for Brazil from 2015 to 2023. Estimations are based on the model estimated by Eq 4 and their related parameters shown in Table 1.
(DOCX)

S4 Table Geometric growth rate (GGR) for calculated nitrogen use efficiency (NUE)–Model fit. (DOCX)

S5 Table Analysis of variance (ANOVA) for the nitrogen fertilizer forecast model. (DOCX)

S6 Table Effect of nitrogen fertilizer consumption on cereal yield for Brazil–Model fit. (DOCX)
