# Supplementary material for: Nitrogen-Use Efficiency, Nitrous Oxide Emissions, and Cereal Production in Brazil: Current Trends and Forecasts
Source: PLoS One. 2015 Aug 7;10(8):e0135234. doi: 10.1371/journal.pone.0135234 (PMC4529221; doi:10.1371/journal.pone.0135234)
Supplement: S1 Table — (DOCX) [file pone.0135234.s001.docx]

**S1 Table. Overview of N fertilizer consumption and associated cereals production in Brazil from 1970 to 2011.**

| *Year* | *Consumption of N fertilizer [1000 tons]* | *Consumption of N fertilizer/cereal^a^ [1000 tons]* | *Cultivated area [million ha]* | *N fertilizer/area [kg/ha^-1^]* | *Cereal production [million ton]* |
| --- | --- | --- | --- | --- | --- |
| 1970 | 278.6 | 147.7 | 16.81 | 8.8 | 23.69 |
| 1971 | 278.3 | 147.5 | 17.67 | 8.3 | 22.80 |
| 1972 | 411.6 | 218.2 | 17.46 | 12.5 | 22.69 |
| 1973 | 347.6 | 184.2 | 16.78 | 11.0 | 23.69 |
| 1974 | 389.2 | 206.3 | 17.99 | 11.5 | 26.20 |
| 1975 | 406.2 | 215.3 | 19.27 | 11.2 | 26.19 |
| 1976 | 498.3 | 264.1 | 21.53 | 12.3 | 31.11 |
| 1977 | 700.5 | 371.3 | 21.26 | 17.5 | 30.89 |
| 1978 | 702.2 | 372.2 | 19.82 | 18.8 | 23.99 |
| 1979 | 778.7 | 412.7 | 20.83 | 19.8 | 27.12 |
| 1980 | 905.5 | 479.9 | 21.05 | 22.8 | 33.19 |
| 1981 | 667.8 | 354.0 | 19.84 | 17.8 | 32.00 |
| 1982 | 642.3 | 340.4 | 21.86 | 15.6 | 33.79 |
| 1983 | 568.0 | 301.1 | 18.05 | 16.7 | 29.16 |
| 1984 | 812.9 | 430.8 | 19.47 | 22.1 | 32.68 |
| 1985 | 834.1 | 442.1 | 19.67 | 22.5 | 35.98 |
| 1986 | 980.9 | 519.9 | 22.37 | 23.2 | 37.26 |
| 1987 | 957.9 | 507.7 | 23.42 | 21.7 | 44.11 |
| 1988 | 815.0 | 431.9 | 23.03 | 18.8 | 42.85 |
| 1989 | 802.5 | 425.3 | 21.93 | 19.4 | 43.89 |
| 1990 | 796.6 | 422.2 | 18.46 | 22.9 | 32.44 |
| 1991 | 830.8 | 440.3 | 19.78 | 22.3 | 36.64 |
| 1992 | 841.3 | 445.9 | 20.53 | 21.7 | 44.02 |
| 1993 | 1085.5 | 575.3 | 18.24 | 31.5 | 43.02 |
| 1994 | 1220.8 | 647.0 | 20.02 | 32.3 | 45.80 |
| 1995 | 1140.1 | 604.3 | 19.71 | 30.7 | 49.59 |
| 1996 | 1250.9 | 663.0 | 17.43 | 38.0 | 42.39 |
| 1997 | 1438.1 | 762.2 | 17.75 | 42.9 | 44.83 |
| 1998 | 1545.5 | 819.1 | 15.74 | 52.0 | 40.69 |
| 1999 | 1660.8 | 880.2 | 17.39 | 50.6 | 47.58 |
| 2000 | 1668.0 | 884.0 | 17.20 | 51.4 | 46.48 |
| 2001 | 1640.0 | 869.2 | 18.09 | 48.0 | 57.08 |
| 2002 | 1834.7 | 972.4 | 17.83 | 54.5 | 50.83 |
| 2003 | 2407.6 | 1276.0 | 19.88 | 64.2 | 67.40 |
| 2004 | 2281.3 | 1209.1 | 20.37 | 59.4 | 63.90 |
| 2005 | 2072.2 | 1098.3 | 19.13 | 57.4 | 55.34 |
| 2006 | 2192.7 | 1162.2 | 18.28 | 63.6 | 58.89 |
| 2007 | 2948.8 | 1562.9 | 19.42 | 80.5 | 69.21 |
| 2008 | 2498.1 | 1324.0 | 20.69 | 64.0 | 79.51 |
| 2009 | 2459.3 | 1303.4 | 19.97 | 65.3 | 70.74 |
| 2010 | 2764.0 | 1464.9 | 18.50 | 79.2 | 74.98 |
| 2011 | 3574.7 | 1894.6 | 19.13 | 99.0 | 77.44 |

^a^The consumption of N fertilizers for cereal production corresponds to 53% of total consumed N fertilizers [33].

Data source: FAOSTAT [11].
