# Supplementary material for: Nitrogen-Use Efficiency, Nitrous Oxide Emissions, and Cereal Production in Brazil: Current Trends and Forecasts
Source: PLoS One. 2015 Aug 7;10(8):e0135234. doi: 10.1371/journal.pone.0135234 (PMC4529221; doi:10.1371/journal.pone.0135234)
Supplement: S2 Table — (DOCX) [file pone.0135234.s002.docx]

**S2 Table.** **Overview of the seven major cereal crops (rice, oats, rye, barley, corn, sorghum, and wheat) production and cultivated area in Brazil from 1970 to 2011.**

| *Year* | *Production [million ton]* | | | | | | | *Cultivated area [1000 ha]* | | | | | | |
| --- | --- | --- | --- | --- | --- | --- | --- | --- | --- | --- | --- | --- | --- | --- |
|  | *Rice* | *Oats* | *Rye* | *Barley* | *Corn* | *Sorghum* | *Wheat* | *Rice* | *Oats* | *Rye* | *Barley* | *Corn* | *Sorghum* | *Wheat* |
| 1970 | 7.55 | 0.03 | 0.02 | 0.03 | 14.22 | 0.00 | 1.84 | 4979.17 | 30.71 | 22.93 | 25.04 | 9858.11 | 0.90 | 1895.25 |
| 1971 | 6.59 | 0.03 | 0.02 | 0.02 | 14.13 | 0.00 | 2.01 | 4764.00 | 34.08 | 23.82 | 23.69 | 10550.49 | 0.90 | 2268.93 |
| 1972 | 6.76 | 0.03 | 0.02 | 0.02 | 14.89 | 0.00 | 0.98 | 4532.93 | 27.70 | 20.42 | 19.83 | 10538.94 | 0.90 | 2319.96 |
| 1973 | 7.16 | 0.04 | 0.02 | 0.01 | 14.19 | 0.25 | 2.03 | 4794.83 | 37.37 | 20.00 | 17.89 | 9923.57 | 146.90 | 1839.39 |
| 1974 | 6.76 | 0.03 | 0.02 | 0.01 | 16.27 | 0.24 | 2.86 | 4664.88 | 34.60 | 22.62 | 16.30 | 10672.45 | 110.97 | 2471.15 |
| 1975 | 7.78 | 0.04 | 0.02 | 0.03 | 16.33 | 0.20 | 1.79 | 5306.27 | 44.79 | 20.86 | 23.73 | 10854.69 | 86.68 | 2931.51 |
| 1976 | 9.76 | 0.04 | 0.01 | 0.06 | 17.75 | 0.28 | 3.22 | 6656.48 | 36.21 | 13.64 | 48.50 | 11117.57 | 121.60 | 3539.89 |
| 1977 | 8.99 | 0.04 | 0.01 | 0.10 | 19.26 | 0.44 | 2.07 | 5992.09 | 39.72 | 9.08 | 93.60 | 11797.41 | 177.64 | 3153.33 |
| 1978 | 7.30 | 0.05 | 0.01 | 0.14 | 13.57 | 0.23 | 2.69 | 5623.52 | 55.55 | 8.19 | 89.42 | 11124.83 | 104.36 | 2811.19 |
| 1979 | 7.60 | 0.06 | 0.01 | 0.10 | 16.31 | 0.12 | 2.93 | 5452.09 | 62.63 | 10.85 | 84.69 | 11318.89 | 71.72 | 3830.54 |
| 1980 | 9.78 | 0.08 | 0.01 | 0.07 | 20.37 | 0.18 | 2.70 | 6243.14 | 75.52 | 12.24 | 72.05 | 11451.29 | 78.21 | 3122.11 |
| 1981 | 8.23 | 0.10 | 0.02 | 0.11 | 21.12 | 0.21 | 2.21 | 6101.77 | 90.23 | 24.31 | 95.62 | 11520.34 | 92.19 | 1920.14 |
| 1982 | 9.73 | 0.06 | 0.00 | 0.10 | 21.84 | 0.23 | 1.83 | 6024.66 | 94.60 | 4.74 | 166.88 | 12619.53 | 122.65 | 2827.93 |
| 1983 | 7.74 | 0.09 | 0.00 | 0.12 | 18.73 | 0.23 | 2.24 | 5108.25 | 95.11 | 4.18 | 120.98 | 10705.98 | 136.29 | 1879.08 |
| 1984 | 9.03 | 0.11 | 0.00 | 0.08 | 21.16 | 0.31 | 1.98 | 5351.47 | 113.72 | 3.78 | 73.19 | 12018.45 | 170.86 | 1741.67 |
| 1985 | 9.02 | 0.17 | 0.01 | 0.17 | 22.02 | 0.27 | 4.32 | 4754.69 | 150.40 | 12.61 | 110.31 | 11798.35 | 170.09 | 2676.73 |
| 1986 | 10.40 | 0.13 | 0.01 | 0.19 | 20.54 | 0.36 | 5.64 | 5590.93 | 123.05 | 5.07 | 102.94 | 12460.13 | 191.01 | 3897.72 |
| 1987 | 10.43 | 0.17 | 0.00 | 0.19 | 26.79 | 0.43 | 6.10 | 6000.02 | 131.50 | 3.02 | 101.66 | 13499.44 | 227.24 | 3454.84 |
| 1988 | 11.81 | 0.13 | 0.00 | 0.13 | 24.75 | 0.29 | 5.75 | 5960.98 | 114.95 | 2.15 | 101.98 | 13181.99 | 190.24 | 3476.29 |
| 1989 | 11.03 | 0.23 | 0.00 | 0.25 | 26.59 | 0.24 | 5.56 | 5254.16 | 195.12 | 3.85 | 113.00 | 12918.98 | 161.09 | 3282.32 |
| 1990 | 7.42 | 0.18 | 0.00 | 0.16 | 21.35 | 0.24 | 3.09 | 3946.69 | 193.20 | 4.40 | 105.07 | 11394.30 | 137.76 | 2680.99 |
| 1991 | 9.49 | 0.23 | 0.01 | 0.11 | 23.62 | 0.26 | 2.92 | 4121.60 | 265.08 | 5.24 | 97.44 | 13063.70 | 173.60 | 2049.46 |
| 1992 | 10.01 | 0.30 | 0.01 | 0.13 | 30.51 | 0.28 | 2.80 | 4687.02 | 284.03 | 6.69 | 66.85 | 13363.60 | 164.30 | 1955.62 |
| 1993 | 10.11 | 0.26 | 0.01 | 0.11 | 30.06 | 0.28 | 2.20 | 4411.32 | 268.02 | 5.63 | 67.06 | 11869.70 | 139.79 | 1482.23 |
| 1994 | 10.54 | 0.26 | 0.00 | 0.09 | 32.49 | 0.32 | 2.10 | 4414.80 | 281.55 | 4.24 | 53.61 | 13748.80 | 165.78 | 1348.85 |
| 1995 | 11.23 | 0.18 | 0.00 | 0.10 | 36.27 | 0.28 | 1.53 | 4373.54 | 165.18 | 2.65 | 69.46 | 13946.30 | 153.96 | 994.73 |
| 1996 | 8.65 | 0.22 | 0.01 | 0.21 | 29.65 | 0.36 | 3.29 | 3253.77 | 160.47 | 5.26 | 84.07 | 11933.80 | 196.75 | 1795.98 |
| 1997 | 8.35 | 0.23 | 0.01 | 0.26 | 32.95 | 0.54 | 2.49 | 3058.13 | 196.80 | 8.22 | 127.63 | 12562.10 | 274.62 | 1521.54 |
| 1998 | 7.72 | 0.21 | 0.01 | 0.30 | 29.60 | 0.59 | 2.27 | 3062.20 | 188.82 | 9.91 | 156.01 | 10585.50 | 331.97 | 1408.85 |
| 1999 | 11.71 | 0.29 | 0.01 | 0.32 | 32.24 | 0.55 | 2.46 | 3813.27 | 218.86 | 6.55 | 137.22 | 11611.48 | 352.39 | 1249.76 |
| 2000 | 11.13 | 0.21 | 0.01 | 0.28 | 32.32 | 0.79 | 1.73 | 3655.29 | 192.98 | 6.71 | 143.78 | 11614.72 | 523.97 | 1065.90 |
| 2001 | 10.18 | 0.34 | 0.01 | 0.30 | 41.96 | 0.91 | 3.37 | 3142.64 | 257.48 | 6.94 | 142.99 | 12330.30 | 486.19 | 1727.39 |
| 2002 | 10.45 | 0.30 | 0.01 | 0.24 | 35.94 | 0.79 | 3.11 | 3145.87 | 254.66 | 4.85 | 147.40 | 11750.90 | 423.60 | 2104.90 |
| 2003 | 10.33 | 0.44 | 0.00 | 0.35 | 48.33 | 1.80 | 6.15 | 3180.86 | 297.08 | 2.74 | 119.22 | 12965.68 | 753.77 | 2560.23 |
| 2004 | 13.28 | 0.46 | 0.00 | 0.40 | 41.79 | 2.16 | 5.82 | 3733.15 | 347.13 | 3.40 | 142.14 | 12410.68 | 931.06 | 2807.22 |
| 2005 | 13.19 | 0.52 | 0.01 | 0.33 | 35.11 | 1.52 | 4.66 | 3915.86 | 367.92 | 4.54 | 144.51 | 11549.43 | 788.19 | 2360.70 |
| 2006 | 11.53 | 0.41 | 0.00 | 0.20 | 42.66 | 1.60 | 2.48 | 2970.92 | 324.00 | 2.93 | 82.18 | 12613.09 | 722.20 | 1560.18 |
| 2007 | 11.06 | 0.24 | 0.00 | 0.24 | 52.11 | 1.44 | 4.11 | 2890.93 | 136.96 | 3.87 | 100.30 | 13767.40 | 662.99 | 1853.22 |
| 2008 | 12.06 | 0.24 | 0.01 | 0.24 | 58.93 | 2.00 | 6.03 | 2850.68 | 117.06 | 4.75 | 79.27 | 14444.58 | 831.35 | 2363.89 |
| 2009 | 12.65 | 0.25 | 0.00 | 0.20 | 50.72 | 1.85 | 5.06 | 2872.04 | 134.04 | 3.63 | 77.45 | 13654.72 | 793.03 | 2430.25 |
| 2010 | 11.24 | 0.40 | 0.00 | 0.28 | 55.36 | 1.53 | 6.17 | 2722.46 | 173.46 | 2.34 | 84.12 | 12678.88 | 661.18 | 2181.57 |
| 2011 | 13.48 | 0.37 | 0.00 | 0.30 | 55.66 | 1.93 | 5.69 | 2752.89 | 172.13 | 2.34 | 88.24 | 13218.89 | 757.41 | 2138.92 |

Data source: FAOSTAT [11].
