# Supplementary material for: Nitrogen-Use Efficiency, Nitrous Oxide Emissions, and Cereal Production in Brazil: Current Trends and Forecasts
Source: PLoS One. 2015 Aug 7;10(8):e0135234. doi: 10.1371/journal.pone.0135234 (PMC4529221; doi:10.1371/journal.pone.0135234)
Supplement: S3 Table — Estimations are based on the model estimated by Eq 4 and their related parameters shown in Table 1. (DOCX) [file pone.0135234.s003.docx]

**S3 Table. Nitrogen use efficiency (NUE) and CO_2_eq emission related to N fertilization from cereal production forecast for Brazil from 2015 to 2023.** Estimations are based on the model estimated by Equation 4 and their related parameters shown in Table 1.

| *Year* | *NUE calculated [%]^b^* | *CO_2_ emissions [Gg CO_2_eq]^a^* |
| --- | --- | --- |
| 2015 | 37.43 (0.0160) | 9105.36 |
| 2016 | 37.67 (0.0162) | 9165.40 |
| 2017 | 38.13 (0.0167) | 9303.02 |
| 2018 | 38.27 (0.0170) | 9452.28 |
| 2019 | 38.43 (0.0174) | 9649.56 |
| 2020 | 38.28 (0.0177) | 9838.10 |
| 2021 | 37.98 (0.0178) | 10003.22 |
| 2022 | 37.73 (0.0180) | 10159.19 |
| 2023 | 37.54 (0.0182) | 10311.37 |
| *Mean* | 37.94 | 9665.28 |
| *Std. Dev.* | 0.361 | 439.56 |
| *CV (%)* | 0.95 | 4.55 |

^a^1Gg = 1000 tons.

^b^The data in brackets are NUE standard deviation.
