# Supplementary material for: Nitrogen-Use Efficiency, Nitrous Oxide Emissions, and Cereal Production in Brazil: Current Trends and Forecasts
Source: PLoS One. 2015 Aug 7;10(8):e0135234. doi: 10.1371/journal.pone.0135234 (PMC4529221; doi:10.1371/journal.pone.0135234)
Supplement: S4 Table — (DOCX) [file pone.0135234.s004.docx]

**S4 Table. Geometric growth rate (GGR) for calculated nitrogen use efficiency (NUE) – Model fit.**

|  | *Coefficient* | | *Bootstrap*  *Std. Err.* | | *P-value* | |
| --- | --- | --- | --- | --- | --- | --- |
| Trend (year) | -0.0242*** | | 0.0022 | | 0.0000 | |
| Constant | 4.4313*** | | 0.0645 | | 0.0000 | |
| *Model fit* | | | | | | |
| *R^2^*: 0.7344; Model *CV*: 4.61 |  |  | | | | |
| *F-value*: 110.58 [0.0000] |  |  | | | | |
| *Durbin stat (DWh)*: 13.01 [0.0003] |  |  | |  | |  |

*Analysis of variance* (ANOVA)

| *Source* | *SS* | *df* | *MS* | *F* | *P-value* |
| --- | --- | --- | --- | --- | --- |
| Model | 3.5991 | 1 | 3.5991 | 110.58 | 0.0010 |
| Residual | 1.3018 | 40 | 0.0325 |  |  |
| Total | 4.9009 | 41 | 0.1195 |  |  |

Asterisks indicate statistically significant differences at 1% (***) test level. The data in brackets for the model fit are P-values.
