# Supplementary material for: Nitrogen-Use Efficiency, Nitrous Oxide Emissions, and Cereal Production in Brazil: Current Trends and Forecasts
Source: PLoS One. 2015 Aug 7;10(8):e0135234. doi: 10.1371/journal.pone.0135234 (PMC4529221; doi:10.1371/journal.pone.0135234)
Supplement: S5 Table — (DOCX) [file pone.0135234.s005.docx]

**S5 Table. Analysis of variance (ANOVA) for the nitrogen fertilizer forecast model.**

| *Source* | *SS* | *df* | *MS* | *F* | *P-value* |
| --- | --- | --- | --- | --- | --- |
| Model | 6.97E+12 | 3 | 2.33E+12 | 175.16 | 0.0000 |
| Residual | 4.91E+11 | 37 | 1.33E+10 |  |  |
| Total | 7.47E+12 | 40 | 1.87E+11 |  |  |
