# Supplementary material for: Nitrogen-Use Efficiency, Nitrous Oxide Emissions, and Cereal Production in Brazil: Current Trends and Forecasts
Source: PLoS One. 2015 Aug 7;10(8):e0135234. doi: 10.1371/journal.pone.0135234 (PMC4529221; doi:10.1371/journal.pone.0135234)
Supplement: S6 Table — (DOCX) [file pone.0135234.s006.docx]

**S6 Table. Effect of nitrogen fertilizer consumption on cereal yield for Brazil – Model fit.**

|  | *Coefficient* | | *Bootstrap*  *Std. Err.* | *P-value* |
| --- | --- | --- | --- | --- |
| N fertilizer | -0.0009*** | | 0.0001 | 0.0001 |
| Constant | 4.8096*** | | 0.1415 | 0.0001 |
| *Model fit* |  | |  |  |
| *R^2^*: 0.9474; Model *CV*: 0.27 |  |  |  |  |
| *F-value*: 936.66 [0.0001] |  |  |  |  |
| *Durbin stat (DWh)*: 0.9748 [0.0500] |  | |  |  |

*Analysis of variance* (ANOVA)

| *Source* | *SS* | *df* | *MS* | *F* | *P-value* |
| --- | --- | --- | --- | --- | --- |
| Model | 68.9878 | 1 | 68.9878 | 936.66 | 0.0001 |
| Residual | 3.8300 | 52 | 0.0737 |  |  |
| Total | 72.8178 | 53 | 1.3739 |  |  |

Asterisks indicate statistically significant differences at 1% (***) test level. The data in brackets for the model fit are P-values.
